# An Ugly Fight

**Published:** 1906-05

**Authors:** 


### EDITORIAL NOTES AND COMMENTS.

The Business office of THE JOURNAL-RECORD is No. 1007-1008 Century Building.

The Editorial office is 318-319-320 The Prudential.

Address all Business communications to Dr. M. B. Hutchins, Mgr.

Make remittances payable to THE ATLANTA JOURNAL-RECORD OF MEDICINE.

On matters pertaining to the Editorial and Original Communications address Dr. Bernard Wolff, Atlanta.

Reprints of original articles will be furnished at cost price. Requests for the same should always be made *on the manuscript.*

We will present, post-paid, on request, to each contributor of an original article, twenty (20) marked copies of THE JOURNAL-RECORD containing such article.

### AN UGLY FIGHT.

We publish herewith literal quotations from the people who have become stirred up by the fight of the *Journal of the American Medical Association,* and other publications, against the patent and proprietary medicines.

Both sides are in part right, and both are in many respects wrong, and raging a nasty warfare which, as extracts show, is catching the doctor between and making him the real sufferer. There was never any good in going to extremes, and these controversies simply engender bitterness and arouse enmity, the effect of which will be hurtful to all concerned and defeat any honest efforts towards needed reforms.

It is not true that all proprietaries are bad. It is not proper for doctors to say too much about self-prescribing or drug-store practice of medicine, for it gives the drug men unfair means of defense. Some proprietaries are better than the average doctor can prescribe and have properly put up by the druggist. As to people going and buying a proprietary direct, so can they do with standard drugs, and, further, one prescription from a doctor is often filled and refilled and even passed on to the neighbors.

On the other hand, if the manufacturers and druggists are to do the prescribing for the sick, why not abolish medical colleges, repeal medical laws, and let every one be a doctor? Why should men devote years to the study of medicine and spend, often, other years of poverty in an effort to heal the sick if the drug people believe that every man should be his own doctor?

As said before, the doctor is the one who is going to suffer through this bitter controversy, and it behooves those who claim to be fighting for the doctor to bear this in mind.

Misrepresentations and false statements are already rife, each side is going to extremes, and unless a more conservative and consistent attitude is taken more harm than good is likely to result. No class should have, or desire, a monopoly, no one of decent honesty should wish to profit by the ills of humanity and none but a ghoul would so profit. Let the fight be fair, stop this unholy war and get together on a basis of "live and let live"—with honor. H.

. . . . . . . . . . .

LEGISLATIVE SCHEMES OF THE AMERICAN MEDICAL ASSOCIATION.*

In nearly if not all of the State Legislatures now in session, bills have been introduced which seek to compel manufacturers of proprietary and patent medicines to make public the formulas and private processes by which their preparations are made. A bill of similar import, dealing with interstate traffic in medicines of this class, has also been proposed in the House of Representatives at Washington.

The large number of these bills, their apparent spontaneity, and the noisy clamor of their advocates, would make it appear that the American people had, all of a sudden, awakened to the realization that they have long been victims of some monstrous

*National Druggist, 1906.

wrong, and that at last, in anger and wrath, they had arisen in their might to wreak revenge upon the authors of their supposed miseries. That such, however, is not the case—that, in fact, there is no real demand from the general public for this legislation, except such as has been, by false representations, created by certain selfish interests, is well-known to all who have followed the current of events in the drug trade for the past three or four years.

In order, therefore, that there may be a clear understanding of the real meaning of this apparently spontaneous movement, and of the influences which are behind it, and to the end that justice be done, not only to the manufacturers whose property it is proposed to destroy, but to the people in general, as well as to the 40,000 retail druggists of the country, of whose total volume of business at least 60 per cent. consists of proprietary medicines, we desire to make this plain statement of facts.

. . . . . . .

There is an organization of doctors known as the American Medical Association. Though it has been in existence for nearly sixty years, it had, up to three or four years ago, less than 5,000 members, out of a total of nearly 150,000 physicians in the entire country.

It was about or a little before that time, it will be remembered, that the great trust movement began, when the people of the United States, all of a sudden, seemed to have tired of doing business on individual lines, and were madly rushing into all kinds of pools, trusts and combinations. It appears that this contagion spread even to portions of the medical profession, for, all at once, a novel and well-conceived plan of organization was adopted by the clique in control of the American Medical Association, the purpose and design of which was to organize all of the doctors in the country into one gigantic medical trust.

There were at that time, and had been for many years, hundreds of county, State and other smaller medical societies, but these were all independent, and had no connection, the one with the other, beyond that bond of sympathy which might naturally be expected to exist among a number of men engaged in the honorable undertaking of trying to uplift the calling or profession to which they had devoted their lives. The new plan proposed to change all this, and contemplated a grand scheme of organization by which all of these small, independent societies should be merged into, or become mere dependents on the American Medical Association, and subject to its laws and regulations. The project was loudly proclaimed and eloquently advocated in the recognized "organ" of the association, known as the *Journal of the American Medical Association;* and the independent medical journals, not yet scenting danger or seeing the ulterior motives behind it, joined heartily in the endeavor to make the scheme a grand success. Paid "organizers" were employed, who visited the local societies, and these, co-operating with the independent journals, were able, in less than three years, to coax or coerce 15,000 physicians into affiliation with the great national association; so that, at the last meeting, it was announced that the membership was in the neighborhood of 20,000, or four times as many as it had been possible after working fifty-five years along the old lines to muster on the rolls of the association.

One of the conditions of membership in the association is the subscribing to the official organ, mentioned above. This journal is published from the sumptuous and spacious headquarters of the association at Chicago, and we learn from the report of the Board of Trustees at the last meeting, that the total income of the journal for the year 1904 was $254,731.45, or probably more than that of a hundred of the smaller independent journals of the profession.

Not satisfied with the one national organ, great and powerful though it is, and resentful of the spirit of revolt which was occasionally manifested in the independent medical press, each of the State societies was induced to establish a journal of its own, ostensibly to publish the proceedings of the association, but really, as it has afterwards developed, to become whippers-in and drummers for the American Medical Association and its official organ. Then there began a systematic campaign against such of the independent journals as had refused to mould their opinions to suit the leaders at Chicago, and members of the association were boldly urged to stop their subscriptions thereto, and to refuse to read any sample copies of the same which might be sent to them. In pleased anticipation of the ultimate success of this effort to stop free discussion and to monopolize the medical press, the *California State Medical Journal* complacently tells us "that the day of the privately-owned medical journal is passing away and its place will be taken by the *Journal of the American Medical Association* and the various State journals."

. . . . . . .

With the amazing growth in membership, as detailed above, with the constantly increasing strength and prosperity of the official organ and its auxiliaries, the officers at Chicago assumed an air of arrogance and insolence, not only towards those physicians who had refused to come into the fold, and to the independent medical press, but also towards the manufacturers of, and dealers in, all kinds of drugs and medicines in the United States. About a year ago these medical autocrats started out on a project to regulate and control the drug industries of the country, and their initial move was the issuance of an edict to the manufacturers of that class of proprietaries intended exclusively for physicians' prescriptions, notifying them that they must forthwith furnish to the Association's Council on Chemistry and Pharmacy, the names and exact quantities of the ingredients which enter into the composition of their several remedies, together with their methods and secret processes of manufacture, all of which should be so complete as to enable the council to "verify them;" that is, to reproduce the preparations, "and to determine their future status from time to time." It was at the same time declared, that unless the manufacturers acceded to these demands, their remedies should be refused admission into a volume which it was proposed to publish, that would contain all remedies of the kind that members of the American Medical Association could "ethically" prescribe or use in their practice. These manufacturers were also warned that failure to comply with the requirements thus glibly set forth, would mean future exclusion from the advertising pages of the "organ," with all the pains and penalties of a general boycott which such exclusion was supposed to carry with it.

It seems, however, that these officials took themselves too seriously. The manufacturers, apparently, did not place as much importance in the official endorsement of the "Council" and in its implied threat of a boycott as these egotists had anticipated. Comparatively few of them seemed willing to take this Council on Chemistry and Pharmacy into their confidence and to entrust to its keeping the valuable trade secrets upon which the prosperity of their business is based, and it was noticed that they began to withdraw their advertisements from the "organ" until, at this writing, the amount of such advertising remaining in the journal is insignificant as compared to that carried in the heyday of its prosperity.

. . . . . . .

The arrant hypocrisy in the pretensions of humanitarianism on the part of these doctor-politicians ought to be apparent to the most guileless individual, but if proofs be needed they shall be forthcoming, and out of their own mouths. We can not, of course, follow them behind the closed doors of their secret deliberations, and it is natural that they should not proclaim their real motives to the public; but occasionally a member, more boldly brutal than his fellows, or one lacking discretion, or for the moment being off his guard, gives the whole snap away and lets the feline out of the bag. And in order to see exactly how they talk when the public ear is not attending, and when, therefore, there is no occasion for assuming a benevolent air, we have taken the trouble to go carefully through a few copies of the *Journal of the American Medical Association* and one or two of its satellites, and we give extracts therefrom, which may help to reveal the real design behind all this agitation against proprietary and patent medicines, and against the druggists who make their living by selling them.

. . . . . . .

To begin with, we will quote from the *Journal of the Association* itself, which is the source of inspiration for all the smaller association and other subservient organs scattered throughout the country. The *Journal,* with its income of a quarter of a million of dollars a year, in its issue of May 6, 1905, urges physicians not even to use one of those proprietary preparations that are especially made for them, and gives as its reason, that "the patient will become acquainted with what the preparation is good for, and will then buy it direct," and consequently some doctor will be cut out of a prescription fee.

Before the Academy of Medicine of New York City, January 18, 1906, proprietary medicines and their bearing on the interests of the association were being discussed, and Dr. Peabody, after considering the difficulty of getting rid of the evil, is reported as declaring, finally, that "we can't prevent people from buying what they want." With this rather pessimistic utterance, Dr. W. Gilman Thompson seemed to take issue, for he proposed as a means to this desired end—that is to prevent people from getting what they want (in other words, to compel them always to go to a doctor and get a prescription before taking medicine)—that:

> The Academy support and work for a bill compelling the labeling of all such preparations with a statement of the character and quantity of the ingredients contained in them, and providing a heavy fine for failure on the part of the manufacturers to comply with it.

The *California State Medical Journal*, quoted above, in its issue for September, 1905, says:

> Ask any pharmacist what will eventually happen if you give a patient a prescription for one of these proprietaries. He will tell you that in due course, the patient, or his wife, or his mother, or his children, or his sisters, or his cousins, or his aunts, or his wife's friends will come into the store and buy more of the same stuff—but without a prescription. In other words, you have lost a patient.

In an article in the *Journal of the American Medical Association*, March 18, 1905, page 894, it is charged "that the druggists are cutting the doctors' throats by selling patent medicines," and an implied threat is made to the druggists in the words that they "ought to see the propriety of not working against the doctors' interests;" that is, by selling patent medicines to the people, and in this way cutting the doctors out of prescription fees. We see no love for the dear people here.

Dr. Horatio C. Wood, Jr., one of the leaders in the present crusade, in the *Journal of the American Medical Association*, June 10, 1905, makes a calculation of the amount spent only in advertising proprietaries, and says that that advertising "represents just so much as coming out of the pockets of the doctors."

In an article in the *Journal of the American Medical Association,* September 9, 1905, page 801, doctors are told that it should be a rule that no proprietary medicine should be delivered to the patient in the original package—this precaution being taken to prevent the purchase of future supplies without a prescription.

Dr. Horatio C. Wood, Jr., again in the *Journal of the American Medical Association,* June 10, 1905, speaking of physicians' proprietaries says: "Indeed the employment of these fancy-named specialties is a direct temptation to self-medication," by which, of course, the doctor is the loser, since it cuts him out of a prescription fee. How altruistic!

### THE MENDACIOUS CAMPAIGN AGAINST DOMESTIC AND PROPRIETARY REMEDIES.

From the *Western Druggist*, Chicago, January, 1906.

In a series of articles appearing in "Collier's" sensational weekly is one on "The Subtle Poisons," which may be said to be typical of the entire series. The writer relates numerous cases of death due directly or indirectly to the use of acetanilid mixtures, and the evil results following the administration of mixtures containing acetanilid. No doubt much that is stated in the article is true; the indiscriminate use of acetanilid mixtures is frequently attended with evil consequences. There is much reason in the contention, therefore, that such indiscriminate use should be discountenanced, but while he is totaling results from the use of drugs why does the writer fail wholly to include in his list of accidents the thousands and tens of thousands of persons injured or of deaths ensuing from the administration of drugs entering into prescriptions? As the symposium on habit-forming drugs which appeared in a recent issue of the *Western Druggist* clearly indicated, nearly every case of the use of habit-forming drugs which came to the attention of the contributors to the symposium commenced originally from a physician's prescription.

To illustrate the utter unfairness and the reckless mendacity which characterizes the treatment of this subject by the Collier writer we republish from the issue of December 2 the following:

CASES AS REPORTED IN COLLIER'S.

"This list of fatalities is made up from statements published in the newspapers. In every case the person who died had taken to relieve a headache or as a bracer a patent medicine containing acetanilid without a doctor's prescription:

Case No. 1—Mrs. Minnie Bishop, Louisville, Ky., October 16, 1903.

Case No. 2—Mrs. Minnie Cusick, New York, N. Y., November 27, 1903.

Case No. 2—Mrs. Julia Ward, New York, N. Y., November 27, 1903.

Case No. 3—Fred P. Stock, Scranton, Pa., December 7, 1903.

Case No. 4—C. Frank Henderson, Toledo, O., December 13, 1903.

Case No. 5—Jacob E. Staley, St. Paul, Mich., February 18, 1904.

Case No. 6—Chas. M. Scott, New Albany, Ind., March 15, 1904.

Case No. 7—Oscar McKinley, Pittsburg, Pa., April 13, 1904.

Case No. 8—Otis Staines, Wabash College, April 13, 1904.

Case No. 9—Mrs. Florence Rumsey, Clinton, Iowa, April 23, 1904.

Case No. 10—Jennie McGee, Philadelphia, Pa., May 26, 1904.

Case No. 11—Mrs. Wm. Mabee, Leoni, Mich., September 9, 1904.

Case No. 12—Mrs. Jacob Friedman, South Bend, Ind., October 19, 1904.

Case No. 13—Libbie North, Rockdale, N. Y., October 26, 1904.

Case No. 14—Margaret Hanahan, Dayton, Ohio, October 29, 1904.

Case No. 15—Samuel Williamson, New York, N. Y., November 21, 1904.

Case No. 16—George Kublisch, St. Louis, Mo., November 27, 1904.

Case No. 17—Robert Breck, St. Louis, Mo., November 27, 1904.

Case No. 18—Mrs. Harry Haven, Oriskany Falls, N. Y., January 17, 1905.

Case No. 19—Mrs. Jennie Whyler, Akron, Ohio, April 3, 1905.

Case No. 20—Mrs. Augusta Strothmann, St. Louis, Mo., June 20, 1905.

Case No. 21—Mrs. Mary A. Bispels, Philadelphia, Pa., July 2, 1905.

Case No. 22—Mrs. Thos. Patterson, Huntington, W. Va., August 15, 1905.

This record may appear startling at first glance, but when it is considered that it covers a period of two years and represents alleged accidents among 80,000,000 of people, there is very little to occasion surprise or alarm. But in order to ascertain the truth in the case exhaustive investgation was made with the following results so far as the investigation has at this time disclosed. A number of cases are still the subject of inquiry and may be reported upon at a later date."

THE FACTS IN THE ABOVE CASES.

Case No. 1—According to the verdict of A. Harris Kelly, coroner, Mrs. Nannie Bishop died October 15, 1903, from cardiac paralysis; contributing cause, accident (acetanilid).

Case No. 2—Mrs. Minnie Cusick and Mrs. Julia Ward, New York City, died, according to Coroner S. Goldenkranz, of ptomaine poisoning, as the result of something they had eaten. The coroner's official record gives the cause of death as "asphyxia, acute gastroenteritis."

Case No. 3—Frank P. Stock, of Scranton, Pa., committed suicide in William Ralder's saloon, 522 Lackawanna avenue, on the afternoon of December 5, 1903, by shooting himself. He left a note to his wife which read as follows: "Darling, the fatal time has come. I am insane and will not be confined behind prison walls. Farewell. Fred." It was rumored at the time that Stock, who was a timekeeper for the D., L. & W. Company, had falsified pay-rolls, and that the discovery of this led to his suicide. The coroner's verdict is that Stock came to his death by a self-inflicted gunshot wound.

Case No. 4—Frank Henderson, Toledo, O., committed suicide in the Jefferson Hotel, December 13. The verdict of Coroner Storz is that "deceased came to his death by reason of suicide by poisoning (morphine)." Henderson wrote a letter to his employers, which was found in the pocket of his coat, asking that his wife, who was at Tiffin, Ohio, be notified.

Case No. 5—There is no "St. Paul, Mich.," to which place Jacob E. Staley is credited. A man by that name died in St. Paul, Minnesota, on February 16, 1904, and was buried in Rutland, Vt. The death certificate signed by the attending physician gives insomnia as chief cause of death, with "overdose sulfonal" as the contributing cause.

Case No. 6—Charles M. Scott, New Albany, Ind. This case is still under investigation, and no facts have yet been secured.

Case No. 7—Oscar McKinley, said to have died at Pittsburg, Pa., April 13, 1904. No death of any person by this name is recorded in Pittsburg, from any cause at or about the time charged.

Case No. 8—Otis Staines, Wabash College, April 13, 1904. This case is still under investigation, and as yet no facts have been secured.

Case No. 9—Mrs. Florence Rumsey, Clinton, Iowa, said to have died April 23, 1904. No death or burial certificate was issued for any person of that name during the year 1904.

Case No. 10—Jennie McGee, Philadelphia, Pa., said to have died on May 26, 1904. There is no such death recorded.

Case No. 11—Mrs. William Mabee, Leoni, Mich., died September 9, 1904. Mrs. Mabee purchased headache medicine in a drug-store in Coldwater, Mich., the preparation being one that the druggist prepared himself. Death, according to Dr. White, was caused by an "overdose of headache tablets, causing paralysis of the heart."

Case No. 12—Mrs. Jacob Friedman, South Bend, Ind. This case is still under investigation.

Case No. 13—Libbie North, Rockdale, N. Y. This case is still under investigation.

Case No. 14—Margaret Hanahan, Dayton, Ohio. Case is still under investigation.

Case No. 15—Samuel Williamson, New York, November 21, 1904. No record of this death can be found.

Case No. 16—George Kublisch, St. Louis, Mo., November 27, 1904. The verdict of the coroner's jury is that death was caused by diabetes and Bright's disease.

Case No. 17—Robert Breck, St. Louis, Mo., November 27, 1904. The death certificate, signed by the coroner, gives as cause of death, "overdose of drug, unknown to jury."

Case No. 18—Mrs. Harry Haven, Oriskany Falls, N. Y., January 17, 1905. The coroner's verdict states that Mrs. Haven, after taking a powder from an open envelope, marked "headache powders," which envelope had laid in a sideboard drawer for some weeks, immediately developed convulsions, which continued for four hours, followed by a semi-comatose condition, deepening into coma, in which she expired seven hours later. The verdict then stated: "A chemical examination of the stomach failed to reveal the presence of strychnia, and a chemical examination of the urine did not disclose the presence of albumin, consequently the cause of convulsions could not be determined. As there was no suspicion of criminality and deceased's friends objected to an examination of the brain, none was made. The coroner concurs in the opinion of the attending physician, that the convulsions were the cause of coma and ultimate dissolution."

Case No. 19—Mrs. Jennie Whyler, Akron, Ohio, April 3, 1905. The verdict of the coroner is that "Mrs. Jennie Whyler came to her death by heart-failure, superinduced by taking headache tablets for the relief of a headache, she being at the time in a weakened state as the result of a recent confinement. The result of taking these tablets produced heart-failure, causing death."

Case No. 20—Mrs. Augusta Strothmann, St. Louis, Mo., June 20, 1905. There is no such death recorded in St. Louis.

Case No. 21—Mrs. Mary A. Bispels, Philadelphia, Pa., July 2, 1905. The verdict of the coroner in this case is that she died of "heart and kidney disease, complicated with poisoning by acetanilid taken in orangeine powders."

Case No. 22—Mrs. Thomas Patterson, Huntington, W. Va., August 15, 1905. This case is still under investigation.

In view of these facts, it is evident that the Collier writer has deliberately falsified in order to make out his case. Clearly the moving impulse of this entire campaign of villification is the desire to promote by lurid sensationalism a purely mercenary notoriety. The effect of these and a flood of simliar writings has been to fill the public mind with alarm. They are doubtless in great measure responsible for bills introduced or to be introduced in various State Legislatures, designed to remedy the alleged evils. Undertaken in the heat of resentment and passion, these bills are certain to take a form attended with great hardship and the greatest peril to every retail druggist who, guilty or innocent, would become the ready victim of ignorant or malicious prosecution. This form of legislation is less an attack upon the proprietary interests of the country, which have shown themselves abundantly able to care for themselves, than upon the retail druggist and his right to dispense and sell his own domestic remedies.

Back of these bills are usually found a coterie of medical men whose professional gospel consists in denunciation of all remedies which do not represent a sufficient "rake-off" in every case for some prescribing doctor. The dispensing of strychnine tablets by medical men is, as statistics at hand conclusively prove, the cause of more accidental deaths, or deaths not accidental, than the total charged to all patent medicines. What remedy is suggested for this evil? The medicine put up by a physician in his practice is as much a domestic or "proprietary" remedy as the one put up by the retail druggist; if the latter is to be labeled "poison," why should not the former? The dispensing-physician curse is vastly more fatal to the people than the so-called domestic remedy or patent medicine curse, and is vastly more in need of prohibitive legislation. Let the medical fraternity do its part toward exterminating the frauds and pretenders which pollute and disgrace their profession; doing this, they will have little time to devote to the regulation of retail druggists, who have shown that they are fully equal to all requirements for effecting their internal reforms.

### A LETTER.

*Atlanta Journal-Record of Medicine, Atlanta, Ga.:*

The bitter and selfish fight being made by the *Journal of the American Medical Association,* some of its satellites, and a few lay papers, on pharmaceutical preparations, has degenerated to a point where misrepresentation and positive mendacity have become their chief stock in trade. While an honest and upright fight was being made for what was supposed by some to be a principle of medical ethics, we were willing to let the matter run its course, knowing that we had little to fear from anything truthfull said of antikamnia or any of the coal-tar preparations. But where lies and other unfair means are used to inflame and mislead the profession and the public, we think it time for us to ask our friends to meet such methods by setting forth the *truth* regarding these attacks.

For instance, *Collier's Weekly* published a list of twenty-two cases of poisoning "from headache remedies," and this was referred to gloatingly by the *Journal of the American Medical Association,* and other medical journals. Yet not one of them all had the grace, fairness or honesty to later publish the fact that a careful investigation of these twenty-two cases by the *Western Druggist* and the *National Druggist* disclosed that only *four* cases were bona fide, the remaining eighteen being cases of death from morphine, strychnine, arsenic, Bright's and other diseases, not in the remotest way connected with headache remedies. One of these trumped-up cases was even found to have been caused by a gunshot wound.

From time to time, apparently inspired reports of deaths or serious illness from the coal-tar pain-relievers appear in the daily press, and investigations of the reports almost invariably show them to be absolutely without a basis of fact, or to have so little truth behind them as to prove beyond peradventure of a doubt that they are inspired by some one whose purpose it is to mislead regarding the safety of these remedies.

The very evident purpose of these reports is to create alarm, *regardless of facts,* touching the coal-tar preparations, so that when the State Legislatures are in session next winter, the *Ladies' Home Journal* bill, and other similar bills classing the coal-tar preparations as poisoons, will pass the various Legislatures.

The coal-tar derivatives appear to be the particular butt of attack by those who are creating the present epidemic of hysteria regarding these remedies, and the most ridiculous and previously unheard of statements are made regarding them. Already bills have been introduced in several State Legislatures classing all coal-tar derivatives as poisons, yet *Collier's,* assisted by the American Medical Association, was, as stated above, able to find only four bona fide cases of untoward effect resulting from *overdoses of all the various coal-tar preparations sold in the United States and Canada.* Yet there have been recorded over 100 cases of quinine blindness, numberless cases of quinine deafness, thousands of cases of skin lesions and other untoward results from small doses of quinine, not to say anything of the numerous deaths caused by it. And quinine is considered a *safe* remedy, and is not mentioned in the Schedule of Poisons referred to in the above mentioned bills.

The coal-tar preparations are not poisons, as that term is usually understood, and every person familiar with medical literature knows—though all may not publicly admit it—that there are more cases of poisoning by almost every other potent drug than by all the various coal-tar preparations combined. This, in spite of the fact that there are probably more doses of the coal-tar pain-relievers taken every day than are taken of any other five drugs.

As to Antikamnia, it has been on the market for over fifteen years, and during all that time not *one* well authenticated case of untoward effect has ever been reported to us where the proper and advised dose was taken. To call a preparation "poison" which never acts even unpleasantly, unless taken in greatly excessive doses, is ridiculous, to say the least. Salt in excessive doses is a poison, and has often produced death. All food contains the most powerful poisons in minute quantities; for instance, prussic acid in fruits, other poisonous substances in meat, fish, eggs, milk, bread, beer, many vegetables, mineral waters, etc. Therefore, these articles of food and drink could more justly be called "poison" than to call a medicine a poison simply because it contains such drugs or a coal-tar preparation. It is essential that a certain prescribed quantity be exceeded to convert any substance into a poison. However, all drugs which are poisonous when given to human beings in doses of a single grain or less, are commonly called "poisons," and these drugs are the substances that people think of when poison is mentioned. To call other drugs "poisons" is misleading, unjust, and is evidence of pure malice.

Of all dishonorable and unprincipled means to injure an article of trade, be it medicine or other substance, the worst is that which libels its character. Millions upon millions of pounds of the coal-tar preparations have been administered in the past twenty years, with injurious results from overdoses so rare as to be the marvel of medical history. The New York Quinine and Chemical Company alone sells over one hundred tons of acetanilid a year. No other potent drug is as safe as these remedies, and those who are attempting, from ulterior motives, to create the impression that they are poisons, are doing an act for which all right-minded people ought to and will condemn them.

"What is a Poison?" is the title of a very able article by Dr. R. G. Eccles, published in *American Medicine* on December 9 and 16, 1905. Every editor of a medical journal should read the said article, now that the attempt is being made to classify the coal-tar preparations as poisons, and he will see the utter absurdity and the plainly dishonest motive behind the work being done to misrepresent these wonderfully safe and efficacious remedies. If the coal-tar preparations are poisons, then quinine, mur. ammonia, chlorate of potash, calomel, sodium bicarb, and the bromides, are virulent poisons in comparison, as are many other drugs which are now considered safe remedies, and which are never referred to as poisons, in the common acceptation of that term.

We write you at length to make plain to you just what is being done to injure the coal-tar preparations, and to show the depths to which seemingly reputable business and professional men will sometimes descend to accomplish a purpose beneficial to themselves.

---

### FEEDING THE BABY.

W. B. Hoag (*American Medicine,* January 6, 1906) says the mother's breast when good gives ideal results. If well fed, the baby is good-natured, comfortable, has firm muscles, a stable nervous system, the bones are normally developed, and he shows a regular increase in weight. Marked deviation in diet, for any considerable period, from that the baby has a right to demand, is bound to produce errors in nutrition. Feeding the baby is not a mechanical process only, simply filling the stomach. Proper growth and development demand definite nutrition. The ability of babies to assimilate food varies; each case presents a personal equation which must be considered. In every questionable case examine the mother's milk. For bottle-feeding, good dairy milk and not "one cow's milk" should form the basis of the food. Set "formulas" are an abomination. Begin with the weakest dilution of cow's milk and increase as the baby assimilates, rather than upset his digestion with a so-called "rational formula" as a starter. Cereal gruels are used successfully in health and disease as diluents of cow's milk. Cane-sugar should be used in preference to milk-sugar. In making working formulas the amount of nutrition the baby receives must be known definitely. Alkalies should not be added regularly to the food; rather secure good milk and have it well prepared and kept. Sterilization or pasteurization should be dependent on quality of milk and time of year. Difficult feeding cases are usually the result of bad management. Quantity at feeding and interval of feeding should be definitely advised. Stomach washing should be employed to promote assimilation. Condensed milk is valuable as a temporary food.—*Medical Age.*

